# Poster Session I - A170 SAFETY AND PROFICIENCY OF ENDOSCOPIC SUBMUCOSAL DISSECTION (ESD) – A 5-YEAR CANADIAN TERTIARY CENTRE SINGLE-OPERATOR EXPERIENCE

**DOI:** 10.1093/jcag/gwaf042.170

**Published:** 2026-02-13

**Authors:** C H Tsai, P J Belletrutti

**Affiliations:** Gastroenterology and Hepatology, University of Calgary, Calgary, AB, Canada; Gastroenterology and Hepatology, University of Calgary, Calgary, AB, Canada

## Abstract

**Background:**

Endoscopic submucosal dissection (ESD) enables en bloc resection of superficial neoplastic lesions, offering superior histologic assessment and lower recurrence rates compared to endoscopic mucosal resection (EMR). However, ESD requires dedicated training, and can be technically demanding and time-intensive, limiting its widespread adoption in Canada.

**Aims:**

This study evaluates the feasibility of running an ESD program by determining the outcomes, learning curve, and safety profile of ESD performed at a Canadian tertiary centre by a single endoscopist after dedicated training.

**Methods:**

Demographic, procedure-specific and outcome data were collected in a prospectively maintained database of all ESDs performed by a single operator in Calgary, Alberta. Data included lesion type, size, resection speed, final pathology, en bloc and curative resection rates, as well as 30-day complications.

**Results:**

Eighty consecutive ESD procedures were performed between November 2019 and August 2025 in the duodenum (n = 10), esophagus (n = 14), stomach (n = 24), and rectum (n = 33). The mean lesion area was 590.1 ± 594.5 mm^2^. Resection speed was evaluated across case quartiles to assess procedural proficiency: 4.85 mm^2^/min (cases 1–20), 10.42 mm^2^/min (cases 21–40), 10.16 mm^2^/min (cases 41–60), and 11.77 mm^2^/min (cases 61–80). Overall, en bloc resection was achieved in 75 cases (93.8%). The en bloc resection rate was maintained across case quartiles: 90% for the first quartile, 100% for the second, 90% for the third, and 95% for the fourth. Final pathology revealed 39 malignant lesions, 10 neuroendocrine tumors (NETs), and 31 benign lesions (adenomas and inflammatory polyps). A curative resection was achieved in 36 of 49 cases (73.5%). Post-procedural complications occurred in 6 patients (7.5%) – GI bleeding in 4, contained rectal perforation in 2 – with no procedure-related mortality.

**Conclusions:**

This single-operator experience demonstrates that ESD is safe and effective outside of high-volume Asian centres, with en bloc and curative resection rates on par with the established literature. Resection speed demonstrated marked improvement with time without compromising safety or efficacy, suggesting that it is feasible from a patient, time, and resource utilization standpoint to run an ESD program in Canada. ESD represents a valuable, minimally invasive alternative to traditional surgery for appropriately selected high-risk lesions.

**Funding Agencies:**

None

